# Exercising the Sanger Sequencing Strategy for Variants Screening and Full-Length Genome of SARS-CoV-2 Virus during Alpha, Delta, and Omicron Outbreaks in Hiroshima

**DOI:** 10.3390/v14040720

**Published:** 2022-03-30

**Authors:** Ko Ko, Kazuaki Takahashi, Shintaro Nagashima, Bunthen E, Serge Ouoba, Toshiro Takafuta, Yoshiki Fujii, Michi Mimori, Fumie Okada, Eisaku Kishita, Kunie Ariyoshi, Md Razeen Ashraf Hussain, Aya Sugiyama, Tomoyuki Akita, Masao Kuwabara, Junko Tanaka

**Affiliations:** 1 Department of Epidemiology, Infectious Disease Control and Prevention, Graduate School of Biomedical and Health Science, Hiroshima University, 1-2-3, Kasumi, Minami-ku, Hiroshima 734-8551, Japan; kko@hiroshima-u.ac.jp (K.K.); ktakaha@hiroshima-u.ac.jp (K.T.); s-nagashima@hiroshima-u.ac.jp (S.N.); d191395@hiroshima-u.ac.jp (B.E.); d192722@hiroshima-u.ac.jp (S.O.); d203067@hiroshima-u.ac.jp (M.R.A.H.); aya-sugiyama@hiroshima-u.ac.jp (A.S.); tomo-akita@hiroshima-u.ac.jp (T.A.); 2 Payment Certification Agency, Ministry of Health, Lot 80, Street 289, Sangkat Boeng Kak Ti Pir, Khan Tuol Kouk, Phnom Penh 12152, Cambodia; 3 Unité de Recherche Clinique de Nanoro (URCN), Nanoro BP18, Burkina Faso; 4 Hiroshima City Funairi Citizens Hospital, 14-11, Funairisaiwaicho, Naka-ku, Hiroshima 730-0844, Japan; toshiro.takafuta@gmail.com; 5 Hiroshima City Institute of Public Health, 4 Chome-1-2, Shoko Center, Nishi-ku, Hiroshima 733-0833, Japan; fujii-yo@city.hiroshima.lg.jp; 6 Hiroshima City Health and Welfare Bureau, 1 Chome-6-34, Kokutaijimachi, Naka-ku, Hiroshima 734-0042, Japan; mimori-m@city.hiroshima.lg.jp; 7 Hiroshima Prefectural Health and Welfare Bureau, 10-52, Motomachi, Naka-ku, Hiroshima 730-8511, Japan; f-okada81372@pref.hiroshima.lg.jp (F.O.); e-kishita74732@pref.hiroshima.lg.jp (E.K.); 8 Hiroshima Prefectural Technology Research Institute, Public Health and Environment Center, 1 Chome-6-29, Minamimachi, Minami-ku, Hiroshima 734-0007, Japan; k-ariyoshi82537@pref.hiroshima.lg.jp; 9 Hiroshima Prefectural Center for Disease Control and Prevention, 10-52, Motomachi, Naka-ku, Hiroshima 730-8511, Japan; m-kuwabaras3471@pref.hiroshima.lg.jp

**Keywords:** screening, Sanger sequencing, variants, SARS-CoV-2, Japan, Omicron

## Abstract

This study aimed to exercise the Sanger sequencing strategy for screening of variants among confirmed COVID-19 cases and validate our strategy against NGS strains in Hiroshima retrieved from GISAID. A total of 660 samples from confirmed COVID-19 cases underwent screening for variants by Sanger-based partial sequencing to the targeted spike gene (nt22,735~nt23,532) using an in-house-developed primer set. The identification of variants was done by unique checkpoints of base nucleotide changes in the targeted spike gene. Moreover, we amplified one full-length genome using Sanger method and an in-house-developed primer library. Using NGS strains of the same sampling period from GISAID, a phylogenetic tree was constructed to examine the distribution pattern of variants in Hiroshima and to validate our Sanger method. The modified primer set provided 100% validation and 99.2% amplification. PANGO Lineage R.1 was detected in late in the third wave, followed by Alpha (B.1.1.7) domination in the fourth wave, Delta (B.1.617.2) domination in the fifth wave, and Omicron (B.1.1.529) domination in the sixth wave, and there was no significant difference in viral copies between variants (*p* = 0.09). The variants showed different transmission patterns, but the distribution of variants is consistent to that shown by the phylogenetic tree. The Sanger method also provided successful amplification of the full-length genome of the SARS-CoV-2 virus. Our Sanger sequencing strategy was useful for the screening of SASR-CoV-2 variants without the need for full-genome amplification. The modified primer set was validated to use universally, which allows an understanding of the variants’ distribution in real time and provides the evidence for policy-making and the formulation or modification of preventive strategies.

## 1. Introduction

As the COVID-19 pandemic is ongoing with the emergence of a variety of variants having distinct mutations bearing different intensities of contagiousness, molecular surveillance is crucial to understand the virus and its characteristics, to adopt effective preventive strategies in a timely manner in response to the variant-dependent infectivity and transmissibility.

The World Health Organization (WHO) monitors and categorizes the SARS-CoV-2 virus into three groups: (a) variant of concern (VOC) if the variant alters its virulence, transmission power, or threatens the effectiveness of preventive measures, (b) variant of interest (VOI) if the variant has the potential for change in its virulence, transmissibility, immune escapes, or diagnostic or therapeutic escapes, and has significant community outbreak, and (c) variant under monitoring (VUM) if the variant is suspected to alter its characteristics but currently has an unclear phenotypic or epidemiological impact that required continuous reassessment [1]. As of January 2022, there are five VOCs (Alpha, Beta, Gamma, Delta, and Omicron variants), two VOIs (Lambda and Mu variants), and three VUMs (PANGO Lineage: B.1.1.318, C.1.2, and B.1.640) [1].

An increasing number of confirmed COVID-19 cases with massive, continuous local or international community outbreaks have occurred everywhere in the world. Every single emergence of a new SARS-CoV-2 variant is associated with the peak and duration of each significant outbreak in the world, providing scientific evidence concerning the viral infectivity, transmissibility, and molecular characteristics of various SARS-CoV-2 variants [2,3,4]. Moreover, the viral replication and the substitution rate driven by the emergence of new SARS-CoV-2 variants subsequently threatens preventive measures and vaccine efficacy, and causes therapeutic or diagnostic difficulty, and delayed or lack of new development of drugs or other therapeutic agents, which collectively delays getting control over the virus and its pandemic.

As the COVID-19 pandemic progresses, a more accurate, universally applicable, and feasible variant screening method is urgently needed to broaden the molecular surveillance, understand the viral evolution, enable early notification of newly emerged variants of concern, and improve the genomic information coverage of the confirmed cases. In Global Initiatives on Sharing All Influenza Data (GISAID: https://www.gisaid.org, accessed on 31 January 2022), only 2.14% of SARS-CoV-2 full-length genomes from all confirmed COVID-19 cases were reported, and next-generation sequencing (NGS) was mainly performed for the full-length genome sequencing and identification of variants. The genomic information coverage among confirmed COVID-19 cases is too low when using NGS. In order to improve variant screening and genomic information coverage, we developed the Sanger sequencing strategy and reported its usefulness for mass screening of SARS-CoV-2 variants using the amplicon-based sequencing targeting a specific partial Spike gene of SARS-CoV-2 [5]. Our Sanger sequencing strategy is validated to identify all types of variants classified by WHO, and the detailed variant screening strategy is described in the previous report [5]. Once the WHO notified of the emergence of the Omicron variant, which has a variety of mutations in the spike gene, especially in our target spike region, in late November 2021, it was necessary to exercise an in-house-developed variant screening strategy to identify the omicron variant. Therefore, this study aimed to exercise the Sanger sequencing strategy for screening of variants among confirmed COVID-19 cases and to validate our screening strategy against NGS-based strains in Hiroshima retrieved from GISAID. Moreover, we amplified the full-length genome of the SARS-CoV-2 virus using the Sanger method and in-house-developed primer library.

## 2. Methods

### 2.1. Subjects

**Figure 1:**
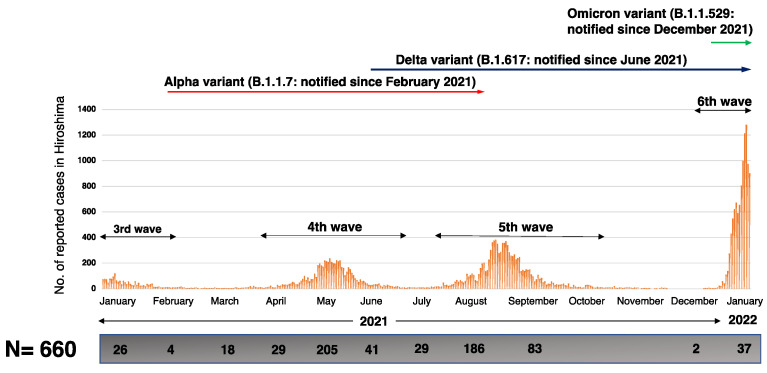
Distribution of study subjects. The upper bar graph represents the number of COVID-19 cases in Hiroshima on a daily basis, which is reported at the Hiroshima Prefectural Health and Welfare Bureau. The number of samples is shown in the box below the bar graph on a monthly basis.

All 660 samples from confirmed COVID-19 cases (514 saliva samples and 146 nasopharyngeal swabs) provided from Hiroshima City Institute of Public Health and Funairi Hospital were included in this study. The abovementioned sample sources are some of the main PCR test centers and COVID-19 treatment centers in Hiroshima city. These samples were collected from the third to sixth outbreak in Hiroshima city from 1 January 2021, to 31 January 2022. All samples were deidentified, and the personal data were not included (Figure 1).

### 2.2. Quantification of SARS-CoV-2 Virus by qRT-PCR

The nucleic acid was extracted from 50 µL of original raw samples using SMITEST EX-R&D (Medical & Biological Laboratories Co., Ltd., Woburn, MA, USA), and the final pallet was dissolved in 25 µL of RNase inhibitor-based water (Thermo Fisher Scientific, Thermo Fisher Scientific, Applied Biosystems, Foster City, CA, USA). The viral titer of SARS-CoV-2 was measured using the previously reported method targeting the nucleocapsid gene of SARS-CoV-2 [6].

### 2.3. Validation of the Modified In-House Primer Set hCoV-Spike-D for Amplification

To validate the efficacy of the in-house-designed primer set for amplification of the specific spike gene of SARS-CoV-2, we used the same samples previously submitted at GISAID [6] (accession number: EPI_ISL_855345 to EPI_ISL_855352) as the control template and performed the amplification by nested RT-PCR using the standard protocol and in-house-designed primer set as shown in Table 1.

### 2.4. Exercising Sanger Sequencing Strategy for SARS-CoV-2 Variants Screening

The extracted nucleic acid was amplified by means of nested RT-PCR as per the previously described method [6]. We used the in-house-designed primer set hCoV-Spike-D for the amplification as shown in Table 1. The amplified products underwent Sanger sequencing for partial genome sequences covering the targeted spike region with SeqStudio sequence Analyzer (Thermo Fisher Scientific, Applied Biosystems, Foster City, CA, USA) and BigDye Terminator v3.1 Cycle Sequencing Kit (Thermo Fisher Scientific, Applied Biosystems, Foster City, CA, USA) and the corresponding primer set as shown in Table 2. First, 2 µL of amplified products was mixed with 4 µL of BigDyeTM Terminator v3.1 Ready Reaction Mix, 2 µL of Sequencing Buffer, 2 µL of 2 µM primer and 10 µL of RNase-free water and incubated at 96 °C for 1 min, followed by 25 cycles of denaturing, annealing, and elongation at 96 °C for 10 s, 50 °C for 5 s, and 60 °C for 4 min. Then, the products were purified by adding a mixture of 90 µL of SAM Solution and 20 µL of BigDye XterminatorTM bead solution. The mixture was thoroughly shaken for 30 min using the micro mixer (MX-4: SANKO JUNYAKU Co., Ltd., Chiyoda-ku Tokyo, Japan). Thereafter, sequencing was performed by SeqStudio Sequence Analyzer following the manufacturer’s instructions.

### 2.5. Identification of SARS-CoV-2 Variants by Unique Checkpoints of Partial Spike Gene

**Figure 2:**
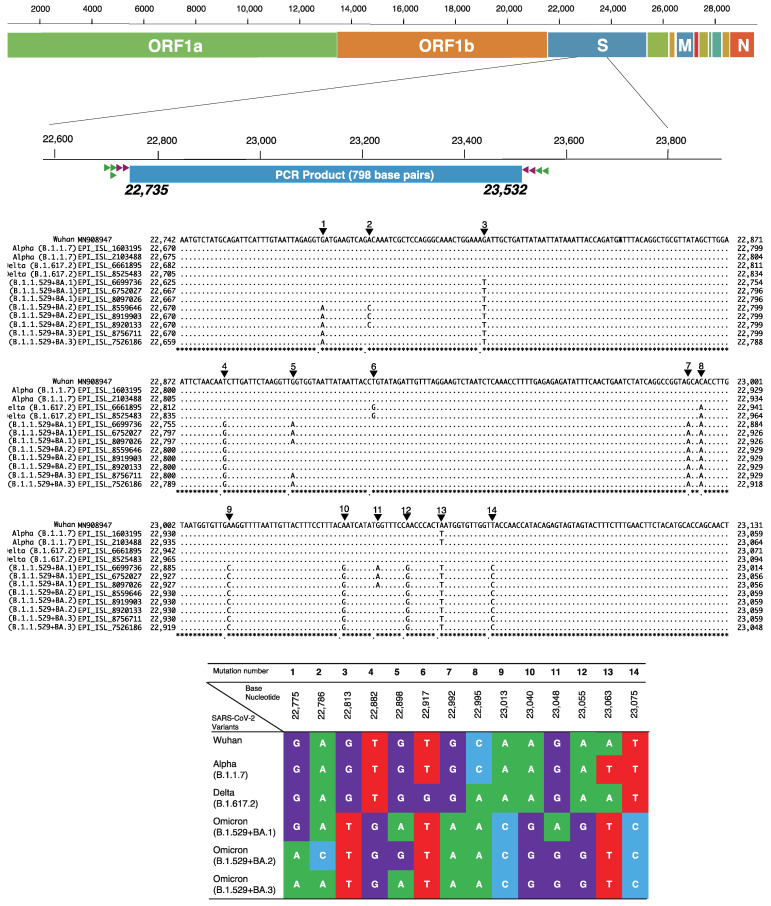
Classification checkpoints in the spike gene for identification of SARS-CoV-2 variants. This figure shows the unique classification checkpoints in the targeted spike gene from base nucleotides 23,735 to 23,532, which covers the part of the spike gene including the receptor-binding domain (RBD) and provides 798 base pairs for PCR. The primers for the first PCR are shown in the green triangle, and those for the second PCR are in the brown triangle. The base nucleotide changes are shown in both alignment sequences and the table box with their respective position. A: Adenine (green color), T: Thymine (red color), G: Guanine (purple color) and C: Cytosine (light blue color).

The variants were then identified by the unique checkpoints of base nucleotide mutation as shown in Figure 2. The GR clade is defined by the GISAID naming system and refers to one of the European clades from which GRY (Alpha variant) and GRA (Omicron variant) were evolved.

### 2.6. Full Genome Sequencing of SARS-CoV-2 Using Sanger Sequencing Method

For the first case infected with Omicron variant admitted at Funairi Hospital, one of the main COVID-19 treatment centers in Hiroshima city, full-length genome sequencing of SARS-CoV-2 was performed using the Sanger sequencing method. We constructed the 16 primer sets in-house, and each primer set was designed to amplify 2~2.5 kilo base pairs of the targeted segments of SARS-CoV-2 (Table 1). The amplification of full-length genome of SARS-CoV-2 was done by two rounds of nested reverse transcriptase polymerase chain rection (nested RT-PCR). The first round of nested RT-PCR used PrimeScriptTM II High Fidelity One Step RT-PCR kit (Takara Bio Inc., Kusatsu, Shiga, Japan), and the reaction protocol consisted of 12.5 µL of 2× One Step High Fidelity Buffer, 2 µL of PrimeSTAR GXL for 1 step RT-PCR, and 0.5 µL of PrimeScript II RT Enzyme Mix, which were all mixed with 2 µL each of sense and antisense primers, 1 µL of distilled water, and 5 µL of template RNA of the sample and then underwent initiation at 45 °C for 10 min and 94 °C for 2 min, 30 cycles of denaturing at 98 °C for 10 s, annealing at 55 °C for 15 s, and elongation at 68 °C for 1 min, followed by final elongation at 68 °C for 7 min. The second round of nested RT-PCR used PrimeSTAR^®^ GXL DNA Polymerase (Takara Bio Inc., Kusatsu, Shiga, Japan), and the reaction protocol contained 10 µL of 5× PrimeSTAR GXL Buffer, 4 µL of dNTP Mixture, 1 µL of PrimeSTAR^®^ GXL DNA Polymerase mixed with 2 µL each of sense and antisense primers, 26 µL of distilled water, and 5 µL of final product from the 1st round of nested RT-PCR. The amplification was processed by the following 30 thermal cycles of denaturing: 98 °C for 10 s, annealing at 55 °C for 15 s, and elongation at 68 °C for 2 min. The amplified products were checked by electrophoresis with 1.5% Prime Gel Agarose and 1 kilo base pair marker. The sequence analysis was performed on the PCR products using the abovementioned Sanger sequencing strategy and the assigned in-house-designed primer library (Table 2).

### 2.7. Phylogenetic Tree of SARS-CoV-2 Full-Genome Sequences in Hiroshima

We collected 2641 SARS-CoV-2 full-length genomes submitted at GISAID from Hiroshima (sampling from January 2021 to January 2022). Additionally, we collected 78 SARS-CoV-2 full-length genomes of Omicron variant submitted from the whole of Japan except Hiroshima, and retrieved Omicron isolates from USA Hawaii, California, and Washington DC using BLAST at GISAID. We also included one SARS-CoV-2 full-length genome of our study (sample ID: FH229) for the phylogenetic analysis. Sequences with many ambiguous sites (N) were removed using GENETYX-Mac (version 21.0.1, GENETYX Corporation, Shibuya-ku, Tokyo, Japan). Then, evolutionary analysis was performed by the neighbor-joining method using Molecular Evolutionary Genetics Analysis (MEGA) version X [7]. Additionally, the concordance in the distribution pattern of SARS-CoV-2 variants between our findings and those reported at GISAID during the same sampling period was evaluated. The comparison was performed using the percent of variants identified by our method against those reported in GISAID on a monthly basis.

## 3. Results

### 3.1. Quantification of SARS-CoV-2 Virus by qRT-PCR

**Figure 3:**
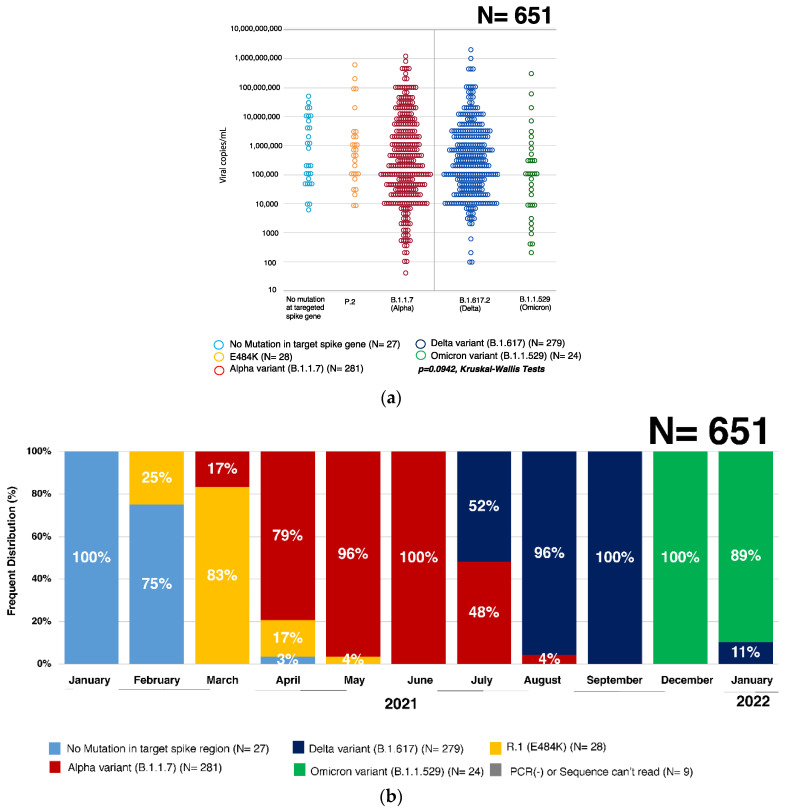
(**a**) The viral titers of SARS-CoV-2 stratified by variants. The scattered plot represents the viral titers detected in all samples of our study, where the blue colored dot represents those samples having no mutation at the targeted spike region, the yellow color represents PANGO Lineage R.1, the red color represents Alpha variant, the dark blue color represents Delta variant, and green represents Omicron variant. The y-axis for the viral titer is expressed in copies per milliliter. (**b**) Percent Distribution of SARS-CoV-2 variants in Hiroshima (January 2021~2022). This figure shows the percent distribution of SARS-CoV-2 variants during the study period in each month. The light blue bar represents those samples having no mutation at the target region, the yellow color represents the PANGO Lineage R.1, the red color represents the Alpha variant, the dark blue is for Delta variant, and green is for Omicron variant.

The viral titer ranged from 5 × 10^1^ to 6 × 10^8^ copies/mL, with the average titer of 2 × 10^7^ copies/mL. There was no significant difference in viral titer between SARS-CoV-2 variants (*p* = 0.0942, Kruskal–Wallis Test) (Figure 3a).

### 3.2. Validation of the Modified In-House Primer Set hCoV-Spike-D for Amplification

We found 100% identity between the reference genome sequences and the new amplified products of all eight samples. There was no amplification-induced mutation using our standard protocol, and it also showed the agreement between Sanger sequencing and the NGS (see Supplementary Figure S1).

### 3.3. Percent Distribution of SARS-CoV-2 Variants in Hiroshima

No mutation was found at the targeted spike gene of SARS-CoV-2 in those samples from January 2021, but in February 2021, 25% had mutation from glutamic acid (E) to lysine (K) at 484 aa (E484K mutation), which is identified as PANGO Lineage R.1. In March 2021, that mutant strain remained dominant (83%) but 17% of cases were infected with Alpha variant (B.1.1.7). From April to August 2021, 79%, 96%, 100%, 48%, and 4% were found to be Alpha variant (B.1.1.7), respectively, in each month, and in July, August, and September 2021, 52%, 96%, and 100%, respectively, were Delta variant (B.1.617.2). In December 2021, the Omicron variant (B.1.1.529) was detected in Hiroshima, and in January 2022, most were infected with the Omicron variant (B.1.1.529) (89%) with the remaining being Delta variant (B.1.1.529) (11%) (Figure 3b and Supplementary Table S1).

The comparison between our findings and those reported at GISAID showed that a similar distribution pattern of SARS-CoV-2 variants circulated in Hiroshima, with some discrepancy in March, April, July and December 2021 (Supplementary Figure S2).

### 3.4. Successful Amplification of SARS-CoV-2 Full-Length Genome Using Combination of Sanger Sequencing Method and In-House-Developed Primer Library

**Figure 4:**
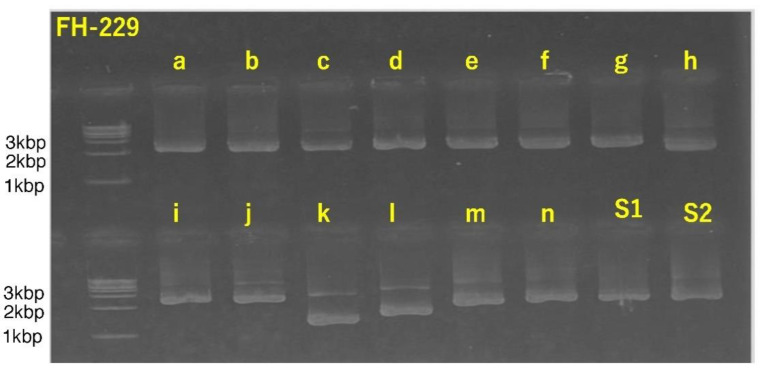
Positive amplification of each segment of SARS-CoV-2 full-length genome from 2nd nested RT-PCR verified by electrophoresis. RT-PCR products of each primer set were described with their respective name a to n, S1 and S2.

We found that the SARS-CoV-2 full-length genome was successfully amplified from the sample FH-229 (EPI_ISL_11505197). The positive amplification was proven with the electrophoresis of the second nested RT-PCR products, as shown in Figure 4. This figure shows the positive amplification of second nested RT-PCR products for the full-length genome of SARS-CoV-2 using the 16 in-house-developed primer sets.

### 3.5. Evolutionary Analysis of SARS-CoV-2 Full-Length Genome Sequences in Hiroshima Retrieved from GISAID

**Figure 5:**
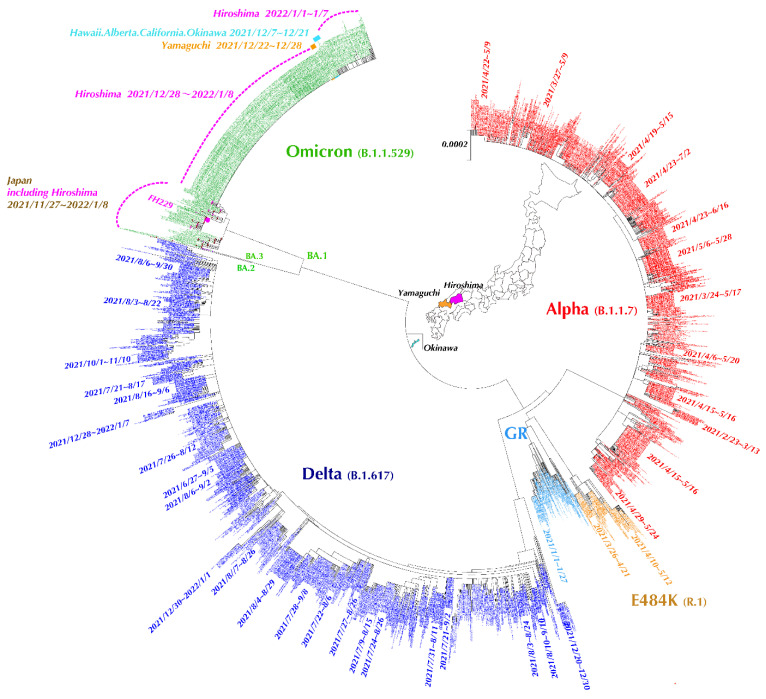
Phylogenetic tree of SARS-CoV-2 isolates detected in Hiroshima. Each variant is shown with a specific color: red for Alpha variant, dark blue for Delta variant, yellow for lineage R.1, green for Omicron, and light blue for GR clade.

This phylogenetic tree includes 2719 NGS strains of the same sampling period retrieved from GISAID, and all those retrieved isolates were from Hiroshima, with additional Omicron strains from Tokyo, Osaka, Okinawa, Yamaguchi, USA Hawaii, and California. Each variant is shown with the specific color: red for Alpha variant, dark blue for Delta variant, yellow for lineage R.1, green for Omicron, and light blue for GR clade. By the phylogenetic tree analysis, many small cluster cases were shown to discretely occur in every outbreak of the COVID-19 pandemic. PANGO Lineage R.1 (E484K mutation) was dominantly found during the third wave, whereas Alpha variant (B.1.1.7) domination was found in the fourth wave, Delta variant (B.1.617.2) domination in the fifth wave, and Omicron variant domination (B.1.1.529) in the sixth wave. (Figure 5) The longest duration of the same cluster of cases transmitted by R.1 variant was 57 days, that by Alpha variant was 55 days, and that by Delta variant was 70 days. The first case of Omicron variant from Funairi Hospital was adjacent to the cluster cases reported from Osaka and Tokyo. However, a large outbreak of Omicron variant occurred in Hiroshima. Using the Basic Local Alignment Search Tool (BLAST) at GISAID combined with phylogenetic tree analysis, those cluster cases were adjacent to those submitted from USA (Hawaii, California, and Washington DC), Canada, Okinawa, and Yamaguchi, showing 100% homology between isolates. (Figure 5)

## 4. Discussion

Based on the previous report of our research group about the development of Sanger sequencing strategy for mass screening of SARS-CoV-2 variants, we modified the primer sets in relation to the newly emerged Omicron variant. The modification included the combination of the previously formulated primer set hCoV-Spike-B and hCoV-Spike-C and the addition of one newly formulated Omicron-adjusted sense primer in first nested RT-PCR. This modification does not mean that the previous protocol and primer set are unable to screen Omicron variant, but the amplification capacity was improved by adding one additional sense primer adjusted to amplify the mutation-rich spike gene of Omicron variant. Therefore, the assigned primer can be used to amplify the targeted spike gene of SARS-CoV-2 enriched with mutations. The primer modification improves the accuracy of screening of all SARS-CoV-2 variants and is useful for examining the newly emerging mutation in the targeted spike gene of SARS-CoV-2.

Using the modified Sanger sequencing strategy, we examined the SARS-CoV-2 variant pattern that circulated and was transmitted during the massive outbreak in Hiroshima. We found that every outbreak was related to the domination of a specific SARS-CoV-2 variant: R.1 variant in the third wave, the Alpha variant (B.1.1.7) in the forth wave, the Delta variant (B.1.617.2) in the fifth wave, and the Omicron variant (B.1.1.529) in the sixth wave. Moreover, each outbreak showed a different wave pattern, differing in the number of cases, outbreak duration, post outbreak interval, and the death rate. Combined with the report from the Hiroshima Prefecture Health Bureau, there was a maximum of 141 newly infected cases per day in the third outbreak (R.1 period), and it lasted for two months (November 2020 to January 2021). In the fourth outbreak (Alpha period), there were maximumly 236 new cases per day (April to June 2021), and the fifth outbreak (Delta period) caused a maximum of 381 new cases per day (July to September 2021). All outbreaks had been controlled after 2 months [8]. The currently ongoing sixth outbreak (Omicron period) started late December 2021, causing a maximum of 1599 new cases per day. All outbreaks showed the transmissibility power of the respective SARS-CoV-2 variants, and the Omicron is the worst and most contagious among all variants that have circulated in Hiroshima.

The percent distribution of variants found in our study was indirectly validated by the phylogenetic tree using 2719 retrieved SARS-CoV-2 full-length genomes and one full-length from our study. All those full-length genomes from GISAID were obtained by NGS. The variant domination pattern was the same as our finding, first with GISAID Clade GR followed by R.1 (E484K mutation) in March, Alpha variant (B.1.1.7) domination from April to June 2021, and then Delta variant domination (B.1.617.2) from July to October 2021. The Omicron variant (B.1.1.259) was detected in December 2021 and later mixed with the Delta variant (B.1.617.2) in January 2022. The Delta variant (B.1.617.2) detected in January 2022 was supposed to have slightly mutated base sequences from those found in 2021. The variant distribution pattern of SARS-CoV-2 in the phylogenetic tree is consistent with that found among 651 patients of our study. Additionally, a similar distribution pattern of SARS-CoV-2 variants was found for both our findings and GISAID, except in March, April, and May 2021 and January 2022. This discrepancy does not indicate the invalidation of Sanger strategy but rather area-dependent different distributions, and sampling bias cannot be ruled out.

Additionally, the phylogenetic tree demonstrates the existence of multiple discrete cluster cases in Hiroshima. The cluster cases varied in each wave, especially between the Alpha domination in forth wave and the Delta domination in fifth wave. Many cluster cases affecting a large number of people infected by the Alpha variant having 100% homology were found to be transmitted for a long period, whereas multiple discrete cluster cases affecting small groups of people infected by the Delta variant having 100% homology were found to be transmitted for a short period, after which time an instant mutation of nucleotide occurred and slightly different strains under the same variant were transmitted, consistent with the existence of variety of subtypes in Delta variant. Moreover, our sample FH-229 was supposed to be transmitted from Tokyo or Osaka. Meanwhile, we noticed the large outbreak in Hiroshima. Therefore, we searched and retrieved the SARS-CoV-2 genome data submitted at GISAID during 28 December 2021~8 January 2022. By phylogenetic tree, 282 isolates with 100% homology were related to this outbreak and were supposed to be transmitted from Yamaguchi prefecture, which is next to Hiroshima Prefecture, and it might have originated from the isolate found in Okinawa and USA Hawaii (most of the submitted isolates from the Okinawa prefecture originated from the US Air Force School of Aerospace Medicine). This phylogenetic tree clearly demonstrates the SARS-CoV-2 variant distribution pattern in Hiroshima, and this pattern probably differed by cities and prefectures.

As the COVID-19 pandemic progresses, there are a total of 370,572,213 confirmed cases with 5,649,390 reported deaths around the world as of 31 January 2022 [9]. Compared to the reported confirmed cases worldwide, 7,946,562 SARS-CoV-2 full-length genomes were submitted at Global Initiatives on Sharing All Influenza Data (GISAID: https://www.gisaid.org) (accessed on 31 January 2022), meaning only 2.14% of sequences from COVID-19 confirmed cases were able to be amplified and translated the genomic information. It is supposed that all the above-mentioned sequences were performed by next-generation sequencing (NGS), which is a parallel sequencing technology offering ultra-high throughput, scalability, and speed but requiring advanced technology, equipment, and special skill, as well as being expensive and time-consuming when completing full-length genomes. Although full-length genome sequencing is the gold standard for molecular surveillance of SARS-CoV-2 variants, it cannot be used as the screening method for variants among large population during the huge outbreak. There are some alternative methods that have been developed for the screening of variants, for example, reverse transcription loop-mediated isothermal amplification (RT-LAMP) and transcription-mediated amplification (TMA) [10], amplicon-based sequencing, single-nucleotide polymorphisms (SNP) assay [11], specific real-time RT-PCR melting curve analysis [12], and virus receptor based electrical biosensor [13]. All alternative methods have their pros and cons over NGS, but the common weak point is a potential diagnostic escapes or misclassification.

Our study has some limitations. As our Sanger strategy mainly focuses on the target spike gene, mutation in other regions can be missed out, but this can be covered by Sanger-based full-length genome sequencing. Recently, GISAID reported on the notification of Delta-Omicron recombinant variant in France (EPI_ISL_10819657); in this case, our variant screening strategy alone would miss out this variant, but a combination of spike and other regions (nucleocapsid gene) sequencing can identify that recombinant variant without the need for full-length genome sequencing.

Our study demonstrated and proved that the Sanger sequencing strategy is a useful tool for screening of SARS-CoV-2 variants in the community, especially where the NGS is not practically accessible or widely used. Moreover, the variants identified by Sanger method are consistent with those identified by the NGS because our Sanger sequencing strategy is based on the highly mutated Spike gene of SARS-CoV-2, which serves as the unique checkpoint for variant classification with high specificity. Additionally, the Sanger method can also be applied to amplify the full-length genomes of SARS-CoV-2.

## 5. Conclusions

Our Sanger sequencing strategy was useful for screening of SARS-CoV-2 variants without the need for full-length genomes amplification. The modified primer set is validated to use universally, which allows an understanding of the variants distribution in time and provides evidence for policy making and formulation or modification of preventive strategies.

## Figures and Tables

**Table 1:** In-house-developed specific primers used for nested RT-PCR of SARS-CoV-2 virus.

	Stage Polarity	Primer Name	Nucleotide Position	Nucleotide Sequence (5′-3′)
hCoV-Spike-D	PCR 1st Sense	22,632S	22,632–22,652	GAATCAGCAACTGTGTTGCTG
	PCR 1st Sense	22,659S	22,659–22,680	CTGTCCTATATAATTCCGCATC
	PCR 1st Sense	22,659S-Omi	22,659–22,680	CTGTCCTATATAATCTCGCACC
	PCR 1st Antisense	SP35AS	23,612–23,631	TGACTAGCTACACTACGTGC
	PCR 1st Antisense	SP36AS	23,577–23,598	TTAGTCTGAGTCTGATAACTAG
	PCR 2nd Sense	22,687S	22,687–22,708	CACTTTTAAGTGTTATGGAGTG
	PCR 2nd Sense	22,712S	22,712–22,734	CCTACTAAATTAAATGATCTCTG
	PCR 2nd Antisense	SP37AS	23,556–23,575	GCATATACCTGCACCAATGG
	PCR 2nd Antisense	SP38AS	23,533–23,554	TATGTCACACTCATATGAGTTG
hCoV-full-a	PCR 1st Sense	45S	37–64	CAACTTTCGATCTCTTGTAGATCTGTTC
	PCR 1st Antisense	a-1-2	1926–1953	TGTAAAACACGCACAGAATTTTGAGCAG
	PCR 2nd Sense	85S	77–102	TTAAAATCTGTGTGGCTGTCACTCGG
	PCR 2nd Antisense	a-2-2	1890–1914	CGGGAGAAAATTGATCGTACAACAC
hCoV-full-b	PCR 1st Sense	b-1-1	1721–1743	GCTTTTGTGGAAACTGTGAAAGG
	PCR 1st Antisense	b-1-2	3878–3902	GCTTAACTTCCTCTTTAGGAATCTC
	PCR 2nd Sense	b-2-1	1763–1785	AAACAAATTGTTGAATCCTGTGG
	PCR 2nd Antisense	b-2-2	3838–3863	CAACTTGCTTTTCACTCTTCATTTCC
hCoV-full-c	PCR 1st Sense	c-1-1	3642–3666	AAGACATTCAACTTCTTAAGAGTGC
	PCR 1st Antisense	c-1-2	5830–5854	AATAGGACCTTTGTATTCTGAGGAC
	PCR 2nd Sense	c-2-1	3683–3706	CAGCACGAAGTTCTACTTGCACCA
	PCR 2nd Antisense	c-2-2	5750–5775	TTATAGTGACCACACTGGTAATTACC
hCoV-full-d	PCR 1st Sense	d-1-1	5572–5597	GTACATGGGCACACTTTCTTATGAAC
	PCR 1st Antisense	d-1-2	7764–7790	CAGCTTTATCAAAGTAAAGATGGATGG
	PCR 2nd Sense	d-2-1	5610–5634	GTGTTCAGATACCTTGTACGTGTGG
	PCR 2nd Antisense	d-2-2	7725–7751	CTGTAACACTATCAACGATGTAAGAAG
hCoV-full-e	PCR 1st Sense	e-1-1	7533–7560	GTACAACTATTGTTAATGGTGTTAGAAG
	PCR 1st Antisense	e-1-2	9729–9753	CTCTTTAGGTAATTACTAAAGAACC
	PCR 2nd Sense	e-2-1	7570–7594	TGTCTATGCTAATGGAGGTAAAGGC
	PCR 2nd Antisense	e-2-2	9692–9719	GCTTTGTGGAAATACAAATGATATAAGC
hCoV-full-f	PCR 1st Sense	f-1-1	9518–9543	TTCCTTATGTCATTCACTGTACTCTG
	PCR 1st Antisense	f-1-2	11,669–11,694	ACACCAAGAGTCAGTCTAAAGTAGCG
	PCR 2nd Sense	f-2-1	9558–9584	ACTCATTCTTACCTGGTGTTTATTCTG
	PCR 2nd Antisense	f-2-2	11,634–11,659	ACAAAAGAGGCCAAAGTAACAAGTAC
hCoV-full-g	PCR 1st Sense	g-1-1	11,459–11,483	TCCATGTGGGCTCTTATAATCTCTG
	PCR 1st Antisense	g-1-2	13,598–13,624	CTTCGTCCTTTTCTTGGAAGCGACAAC
	PCR 2nd Sense	g-2-1	11,494–11,518	CTACTCAGGTGTAGTTACAACTGTC
	PCR 2nd Antisense	g-2-2	13,554–13,579	TAGCAAAACCAGCTACTTTATCATTG
hCoV-full-h	PCR 1st Sense	h-1-1	13,384–13,407	CGGTATGTGGAAAGGTTATGGCTG
	PCR 1st Antisense	h-1-2	15,569–15,595	ACTTATCGGCAATTTTGTTACCATCAG
	PCR 2nd Sense	h-2-1	13,418–13,441	CAACTCCGCGAACCCATGCTTCAG
	PCR 2nd Antisense	h-2-2	15,538–15,563	AAAAGTGCATTAACATTGGCCGTGAC
hCoV-full-i	PCR 1st Sense	i-1-1	15,334–15,359	ATTATGGCCTCACTTGTTCTTGCTCG
	PCR 1st Antisense	i-1-2	17,506–17,533	CTATAGTTTTCATAAGTCTACACACTCC
	PCR 2nd Sense	i-2-1	15,380–15,406	GCTTGTCACACCGTTTCTATAGATTAG
	PCR 2nd Antisense	i-2-2	17,453–17,478	CTTAGTTAGCAATGTGCGTGGTGCAG
hCoV-full-j	PCR 1st Sense	j-1-1	17,278–17,304	GTGAATTCAACATTAGAACAGTATGTC
	PCR 1st Antisense	j-1-2	19,477–19,502	GCATGATGTCTACAGACAGCACCACC
	PCR 2nd Sense	j-2-1	17,317–17,341	AATGCATTGCCTGAGACGACAGCAG
	PCR 2nd Antisense	j-2-2	19,448–19,473	ATTGCAACGTGTTATACACGTAGCAG
hCoV-full-k	PCR 1st Sense	k-1-1	19,239–19,264	ATTTGACACTAGAGTGCTATCTAACC
	PCR 1st Antisense	k-1-2	21,423–21,449	TCTTTTAAAGACATAACAGCAGTACCC
	PCR 2nd Sense	k-2-1	19,275–19,300	TGGTTGTGATGGTGGCAGTTTGTATG
	PCR 2nd Antisense	k-2-2	21,386–21,412	GGGGAAATTTACTCATGTCAAATAAAG
hCoV-full-l	PCR 1st Sense	l-1-1	25,369–25,396	ATTACATTACACATAAACGAACTTATGG
	PCR 1st Antisense	l-1-2	27,416–27,442	GCTCACAAGTAGCGAGTGTTATCAGTG
	PCR 2nd Sense	l-2-1	25,398–25,424	TTTGTTTATGAGAATCTTCACAATTGG
	PCR 2nd Antisense	l-2-2	27,358–27,384	ATCAATCTCCATTGGTTGCTCTTCATC
hCoV-full-m	PCR 1st Sense	m-1-1	27,175–27,201	CTTTGCTTGTACAGTAAGTGACAACAG
	PCR 1st Antisense	m-1-2	28,771–28,797	CTTCTGCGTAGAAGCCTTTTGGCAATG
	PCR 2nd Sense	m-2-1	27,211–27,236	CTCGTTGACTTTCAGGTTACTATAGC
	PCR 2nd Antisense	m-2-2	28,737–28,763	TTGAGGAAGTTGTAGCACGATTGCAGC
hCoV-full-n	PCR 1st Sense	n-1-1	28,539–28,563	AGAGCTACCAGACGAATTCGTGGTG
	PCR 1st Antisense	n-1-2	29,854–29,882	TTTTTTTTTTTGTCATTCTCCTAAGAAGC
	PCR 2nd Sense	n-2-1	28,595–28,621	ATGGTATTTCTACTACCTAGGAACTGG
	PCR 2nd Antisense	n-2-2	29,826–29,852	ATTAAAATCACATGGGGATAGCACTAC
hCoV-full-S1	PCR 1st Sense	20,963S	20,955–20,980	TCTTAATGACTTTGTCTCTGATGCAG
	PCR 1st Sense	21,018S	21,010–21,034	GTACATACAGCTAATAAATGGGATC
	PCR 1st Antisense	23,504AS	23,496–23,518	CCCTATTAAACAGCCTGCACGTG
	PCR 2nd Sense	21,050S	21,042–21,065	TAGTGATATGTACGAC CCTAAGAC
	PCR 2nd Sense	21,018S	21,010–21,034	GTACATACAGCTAATAAATGGGATC
	PCR 2nd Antisense	23,461AS	23,453–23,476	TGTAGAATAAACACGCCAAGTAGG
hCoV-full-S2	PCR 1st Sense	23,254S	23,246–23,270	TTYCAACAATTTGGCAGAGACATTG
	PCR 1st Antisense	25,666AS	25,658–25,681	CGAGCAAAAGGTGTGAGTAAACTG
	PCR 2nd Sense	23,283S	23,275–23,297	CACTACTGATGCTGTCCGTGATC
	PCR 2nd Antisense	25,619AS	25,611–25,633	AAACAAAGTGAACACCCTTGGAG

**Table 2:** In-house-developed primer library used for sequence analysis of SARS-CoV-2 virus.

	Primer Name	Nucleotide Sequence (5′-3′)
hCoV-Spike-D	22712-S	CCTACTAAATTAAATGATCTCTG
	Spike-AS	CACCAGCAACTGTTTGTGGAC
hCoV-full-a	85-S	TTAAAATCTGTGTGGCTGTCACTCGG
	a-2-2	CGGGAGAAAATTGATCGTACAACAC
	a-1-Seq	TTAGGCGACGAGCTTGGCACTG
hCoV-full-b	b-2-1	AAACAAATTGTTGAATCCTGTGG
	b-2-2	CAACTTGCTTTTCACTCTTCATTTCC
	b-1-Seq	GTTAAATCCAGAGAAGAAACTGG
	b-S-1	ATTGGCTTCACATATGTATTG
	b-AS-1	CTTCAATAGTCTGAACAACTG
hCoV-full-c	c-2-1	CAGCACGAAGTTCTACTTGCACCA
	c-2-2	TTATAGTGACCACACTGGTAATTACC
	c-1-Seq	TGGAATTTGCGAGAAATGCTTG
	c-S-1	AACAACTGTAGCGTCACTTATC
hCoV-full-d	d-2-1	GTGTTCAGATACCTTGTACGTGTGG
	d-2-2	CTGTAACACTATCAACGATGTAAGAAG
	d-1-Seq	ATTGTTTGGCATGTTAACAATG
	d-S1	CTTCAAGAGAGCTTAAAGTTAC
	d-AS-1	TAAGCCAAAAGCAGTTAAATCC
hCoV-full-e	e-2-1	TGTCTATGCTAATGGAGGTAAAGGC
	e-2-2	GCTTTGTGGAAATACAAATGATATAAGC
	e-S-1	AATGTGTCCTTAGACAATGTC
	e-AS-1	TGATCTTTCACAAGTGCCGTG
hCoV-full-f	f-2-1	ACTCATTCTTACCTGGTGTTTATTCTG
	f-2-2	ACAAAAGAGGCCAAAGTAACAAGTAC
	f-1-Seq	GAAGATTTACTCATTCGTAAGTC
	f-S-1	AGTAACTTGTGGTACAACTAC
	f-AS-1	TACGCATCACCCAACTAGCAG
hCoV-full-g	g-2-1	CTACTCAGGTGTAGTTACAACTGTC
	g-2-2	TAGCAAAACCAGCTACTTTATCATTG
	g-1-Seq	AGCAGGCTGTTGCTAATGGTG
	g-S1	TTTCCATGCAGGGTGCTGTAG
	g-AS-1	AATTGGCAGGCACTTCTGTTG
hCoV-full-h	h-2-1	CAACTCCGCGAACCCATGCTTCAG
	h-2-2	AAAAGTGCATTAACATTGGCCGTGAC
	h-1-Seq	TCAAGATCTCAATGGTAACTGG
	hS1	ATTGTTGGTGTACTGACATTAG
	hAS1	CTTGATCCTCATAACTCATTG
hCoV-full-i	i-2-1	GCTTGTCACACCGTTTCTATAGATTAG
	i-2-2	CTTAGTTAGCAATGTGCGTGGTGCAG
	i-1-Seq	GTCTTTAGCTATAGATGCTTACC
	i-S-1	CAGATGGTACACTTATGATTG
	i-AS-1	ATAGTGCTCTTGTGGCACTAG
hCoV-full-j	j-2-1	AATGCATTGCCTGAGACGACAGCAG
	j-2-2	ATTGCAACGTGTTATACACGTAGCAG
	j-1-Seq	GAAATTCCACGTAGGAATGTGG
	j-S-1	TTGATTCATCACAGGGCTCAG
	j-AS-1	CAGTTCATCACCAATTATAGG
hCoV-full-k	k-2-1	TGGTTGTGATGGTGGCAGTTTGTATG
	k-2-2	GGGGAAATTTACTCATGTCAAATAAAG
	k-1-Seq	TTCTATGACTGACATAGCCAAG
	k-S-1	GCAACATTAAACCAGTACCAG
	k-AS-1	CAACTCCTTTATCAGAACCAG
hCoV-full-l	l-2-1	TTTGTTTATGAGAATCTTCACAATTGG
	l-2-2	ATCAATCTCCATTGGTTGCTCTTCATC
	l-1-Seq	CTCAATTGAGTACAGACACTGG
hCoV-full-m	m-2-1	CTCGTTGACTTTCAGGTTACTATAGC
	m-2-2	TTGAGGAAGTTGTAGCACGATTGCAGC
	m-1-Seq	GGTTCTCACTTGAACTGCAAG
hCoV-full-n	n-2-1	ATGGTATTTCTACTACCTAGGAACTGG
	n-2-2	ATTAAAATCACATGGGGATAGCACTAC
hCoV-full-S1	21018S	GTACATACAGCTAATAAATGGGATC
	23461AS	TGTAGAATAAACACGCCAAGTAGG
	S1-1-Seq	CACTGACACCACCAAAGAACAT
	S1-S1	GTTAACAACTAAACGAACAATG
	S1-S2	GTTCAGAGTTTATTCTAGTGCG
	S1-S3	GGTGCTGCAGCTTATTATGTGG
hCoV-full-S2	23283S	CACTACTGATGCTGTCCGTGATC
	25619AS	AAACAAAGTGAACACCCTTGGAG
	S2-2-Seq	AACGGCCTTACTGTTTGCCACC
	S2-S1	ATTCAACTGAATGCAGCAATC
	S2-S2	AACTTAGCTCCAAATTTGGTGC

## Data Availability

All data used in this study are fully described in the figure and tables. All partial genomes sequence data of SARS-CoV-2 included in this study are deposited at GenBank and one SARS-CoV-2 full-length genome (FH-229) was deposited at GISAID with accession no. EPI_ISL_11505197 if there is any trouble in accessing the data, the sequences are available from the corresponding author upon the reasonable request.

## Supplementary Materials

The following supporting information can be downloaded at: https://www.mdpi.com/article/10.3390/v14040720/s1, Figure S1: The validity of amplification and sequencing between Sanger Vs Next Generation Sequencing (NGS); Figure S2: The distribution of SARS-CoV-2 variants between Sanger and GISAID (January 2021–2022); Table S1: Distribution of SARS-CoV-2 variants using Sanger Method (January 2021–2022).
