# Supplementary material for: Exercising the Sanger Sequencing Strategy for Variants Screening and Full-Length Genome of SARS-CoV-2 Virus during Alpha, Delta, and Omicron Outbreaks in Hiroshima
Source: Viruses. 2022 Mar 30;14(4):720. doi: 10.3390/v14040720 (PMC9030034; doi:10.3390/v14040720)
Supplement: Supplementary file 1 [file viruses-14-00720-s001.zip › Supplementary Figure S1.pdf]

22791 ▼

```
hCoV-19_Japan_Hiro66017_2020_EPI_ISL_855345_2020-03-14    1 TCGCTCCAGGGCAAACTGGAAAGATTGCTGATTATAATTATAAATTACCAGATGATTTTACAGGCTGCGTTATAGCTTGGAATTCTAACAATCTTGATTCTAAGGTTGGTGGTAATTATAATTACCTGTATAGATTGTTTAGGAAGTCTA  150
Hiro66017_14-03-2020                                      1 ......................................................................................................................................................  150

hCoV-19_Japan_Hiro99262_2020_EPI_ISL_855346_2020-04-11    1 TCGCTCCAGGGCAAACTGGAAAGATTGCTGATTATAATTATAAATTACCAGATGATTTTACAGGCTGCGTTATAGCTTGGAATTCTAACAATCTTGATTCTAAGGTTGGTGGTAATTATAATTACCTGTATAGATTGTTTAGGAAGTCTA  150
Hiro99262_11-04-2020                                      1 ......................................................................................................................................................  150

hCoV-19_Japan_Hiro99268_2020_EPI_ISL_855347_2020-04-11    1 TCGCTCCAGGGCAAACTGGAAAGATTGCTGATTATAATTATAAATTACCAGATGATTTTACAGGCTGCGTTATAGCTTGGAATTCTAACAATCTTGATTCTAAGGTTGGTGGTAATTATAATTACCTGTATAGATTGTTTAGGAAGTCTA  150
Hiro99268_11-04-2020                                      1 ......................................................................................................................................................  150

hCoV-19_Japan_Hiro94253_2020_EPI_ISL_855348_2020-04-20    1 TCGCTCCAGGGCAAACTGGAAAGATTGCTGATTATAATTATAAATTACCAGATGATTTTACAGGCTGCGTTATAGCTTGGAATTCTAACAATCTTGATTCTAAGGTTGGTGGTAATTATAATTACCTGTATAGATTGTTTAGGAAGTCTA  150
Hiro94253_20-04-2020                                      1 ......................................................................................................................................................  150

hCoV-19_Japan_Hiro96977_2020_EPI_ISL_855349_2020-07-27    1 TCGCTCCAGGGCAAACTGGAAAGATTGCTGATTATAATTATAAATTACCAGATGATTTTACAGGCTGCGTTATAGCTTGGAATTCTAACAATCTTGATTCTAAGGTTGGTGGTAATTATAATTACCTGTATAGATTGTTTAGGAAGTCTA  150
Hiro96977_27-07-2020                                      1 ......................................................................................................................................................  150

hCoV-19_Japan_Hiro97166_2020_EPI_ISL_855350_2020-07-29    1 TCGCTCCAGGGCAAACTGGAAAGATTGCTGATTATAATTATAAATTACCAGATGATTTTACAGGCTGCGTTATAGCTTGGAATTCTAACAATCTTGATTCTAAGGTTGGTGGTAATTATAATTACCTGTATAGATTGTTTAGGAAGTCTA  150
Hiro97166_29-07-2020                                      1 ......................................................................................................................................................  150

hCoV-19_Japan_Hiro97237_2020_EPI_ISL_855351_2020-07-30    1 TCGCTCCAGGGCAAACTGGAAAGATTGCTGATTATAATTATAAATTACCAGATGATTTTACAGGCTGCGTTATAGCTTGGAATTCTAACAATCTTGATTCTAAGGTTGGTGGTAATTATAATTACCTGTATAGATTGTTTAGGAAGTCTA  150
Hiro97237_30-07-2020                                      1 ......................................................................................................................................................  150

hCoV-19_Japan_Hiro97618_2020_EPI_ISL_855352_2020-08-03    1 TCGCTCCAGGGCAAACTGGAAAGATTGCTGATTATAATTATAAATTACCAGATGATTTTACAGGCTGCGTTATAGCTTGGAATTCTAACAATCTTGATTCTAAGGTTGGTGGTAATTATAATTACCTGTATAGATTGTTTAGGAAGTCTA  150
Hiro97618_03-08-2020                                      1 ......................................................................................................................................................  150
                                                            ******************************************************************************************************************************************************

hCoV-19_Japan_Hiro66017_2020_EPI_ISL_855345_2020-03-14  151 ATCTCAAACCTTTTGAGAGAGATATTTCAACTGAAATCTATCAGGCCGGTAGCACACCTTGTAATGGTGTTGAAGGTTTTAATTGTTACTTTCCTTTACAATCATATGGTTTCCAACCCACTAATGGTGTTGGTTACCAACCATACAGAG  300
Hiro66017_14-03-2020                                    151 ......................................................................................................................................................  300

hCoV-19_Japan_Hiro99262_2020_EPI_ISL_855346_2020-04-11  151 ATCTCAAACCTTTTGAGAGAGATATTTCAACTGAAATCTATCAGGCCGGTAGCACACCTTGTAATGGTGTTGAAGGTTTTAATTGTTACTTTCCTTTACAATCATATGGTTTCCAACCCACTAATGGTGTTGGTTACCAACCATACAGAG  300
Hiro99262_11-04-2020                                    151 ......................................................................................................................................................  300

hCoV-19_Japan_Hiro99268_2020_EPI_ISL_855347_2020-04-11  151 ATCTCAAACCTTTTGAGAGAGATATTTCAACTGAAATCTATCAGGCCGGTAGCACACCTTGTAATGGTGTTGAAGGTTTTAATTGTTACTTTCCTTTACAATCATATGGTTTCCAACCCACTAATGGTGTTGGTTACCAACCATACAGAG  300
Hiro99268_11-04-2020                                    151 ......................................................................................................................................................  300

hCoV-19_Japan_Hiro94253_2020_EPI_ISL_855348_2020-04-20  151 ATCTCAAACCTTTTGAGAGAGATATTTCAACTGAAATCTATCAGGCCGGTAGCACACCTTGTAATGGTGTTGAAGGTTTTAATTGTTACTTTCCTTTACAATCATATGGTTTCCAACCCACTAATGGTGTTGGTTACCAACCATACAGAG  300
Hiro94253_20-04-2020                                    151 ......................................................................................................................................................  300

hCoV-19_Japan_Hiro96977_2020_EPI_ISL_855349_2020-07-27  151 ATCTCAAACCTTTTGAGAGAGATATTTCAACTGAAATCTATCAGGCCGGTAGCACACCTTGTAATGGTGTTGAAGGTTTTAATTGTTACTTTCCTTTACAATCATATGGTTTCCAACCCACTAATGGTGTTGGTTACCAACCATACAGAG  300
Hiro96977_27-07-2020                                    151 ......................................................................................................................................................  300

hCoV-19_Japan_Hiro97166_2020_EPI_ISL_855350_2020-07-29  151 ATCTCAAACCTTTTGAGAGAGATATTTCAACTGAAATCTATCAGGCCGGTAGCACACCTTGTAATGGTGTTGAAGGTTTTAATTGTTACTTTCCTTTACAATCATATGGTTTCCAACCCACTAATGGTGTTGGTTACCAACCATACAGAG  300
Hiro97166_29-07-2020                                    151 ......................................................................................................................................................  300

hCoV-19_Japan_Hiro97237_2020_EPI_ISL_855351_2020-07-30  151 ATCTCAAACCTTTTGAGAGAGATATTTCAACTGAAATCTATCAGGCCGGTAGCACACCTTGTAATGGTGTTGAAGGTTTTAATTGTTACTTTCCTTTACAATCATATGGTTTCCAACCCACTAATGGTGTTGGTTACCAACCATACAGAG  300
Hiro97237_30-07-2020                                    151 ......................................................................................................................................................  300

hCoV-19_Japan_Hiro97618_2020_EPI_ISL_855352_2020-08-03  151 ATCTCAAACCTTTTGAGAGAGATATTTCAACTGAAATCTATCAGGCCGGTAGCACACCTTGTAATGGTGTTGAAGGTTTTAATTGTTACTTTCCTTTACAATCATATGGTTTCCAACCCACTAATGGTGTTGGTTACCAACCATACAGAG  300
Hiro97618_03-08-2020                                    151 ......................................................................................................................................................  300
                                                            ******************************************************************************************************************************************************

hCoV-19_Japan_Hiro66017_2020_EPI_ISL_855345_2020-03-14  301 TAGTAGTACTTTCTTTTGAACTTCTACATGCACCAGCAACTGTTTGTGGACCTAAAAAGTCTACTAATTTGGTTAAAAACAAATGTGTCAATTTCAACTTCAATGGTTTAACAGGCACAGGTGTTCTTACTGAGTCTAACAAAAAGTTTC  450
Hiro66017_14-03-2020                                    301 ......................................................................................................................................................  450

hCoV-19_Japan_Hiro99262_2020_EPI_ISL_855346_2020-04-11  301 TAGTAGTACTTTCTTTTGAACTTCTACATGCACCAGCAACTGTTTGTGGACCTAAAAAGTCTACTAATTTGGTTAAAAACAAATGTGTCAATTTCAACTTCAATGGTTTAACAGGCACAGGTGTTCTTACTGAGTCTAACAAAAAGTTTC  450
Hiro99262_11-04-2020                                    301 ......................................................................................................................................................  450

hCoV-19_Japan_Hiro99268_2020_EPI_ISL_855347_2020-04-11  301 TAGTAGTACTTTCTTTTGAACTTCTACATGCACCAGCAACTGTTTGTGGACCTAAAAAGTCTACTAATTTGGTTAAAAACAAATGTGTCAATTTCAACTTCAATGGTTTAACAGGCACAGGTGTTCTTACTGAGTCTAACAAAAAGTTTC  450
Hiro99268_11-04-2020                                    301 ......................................................................................................................................................  450

hCoV-19_Japan_Hiro94253_2020_EPI_ISL_855348_2020-04-20  301 TAGTAGTACTTTCTTTTGAACTTCTACATGCACCAGCAACTGTTTGTGGACCTAAAAAGTCTACTAATTTGGTTAAAAACAAATGTGTCAATTTCAACTTCAATGGTTTAACAGGCACAGGTGTTCTTACTGAGTCTAACAAAAAGTTTC  450
Hiro94253_20-04-2020                                    301 ......................................................................................................................................................  450

hCoV-19_Japan_Hiro96977_2020_EPI_ISL_855349_2020-07-27  301 TAGTAGTACTTTCTTTTGAACTTCTACATGCACCAGCAACTGTTTGTGGACCTAAAAAGTCTACTAATTTGGTTAAAAACAAATGTGTCAATTTCAACTTCAATGGTTTAACAGGCACAGGTGTTCTTACTGAGTCTAACAAAAAGTTTC  450
Hiro96977_27-07-2020                                    301 ......................................................................................................................................................  450

hCoV-19_Japan_Hiro97166_2020_EPI_ISL_855350_2020-07-29  301 TAGTAGTACTTTCTTTTGAACTTCTACATGCACCAGCAACTGTTTGTGGACCTAAAAAGTCTACTAATTTGGTTAAAAACAAATGTGTCAATTTCAACTTCAATGGTTTAACAGGCACAGGTGTTCTTACTGAGTCTAACAAAAAGTTTC  450
Hiro97166_29-07-2020                                    301 ......................................................................................................................................................  450

hCoV-19_Japan_Hiro97237_2020_EPI_ISL_855351_2020-07-30  301 TAGTAGTACTTTCTTTTGAACTTCTACATGCACCAGCAACTGTTTGTGGACCTAAAAAGTCTACTAATTTGGTTAAAAACAAATGTGTCAATTTCAACTTCAATGGTTTAACAGGCACAGGTGTTCTTACTGAGTCTAACAAAAAGTTTC  450
Hiro97237_30-07-2020                                    301 ......................................................................................................................................................  450

hCoV-19_Japan_Hiro97618_2020_EPI_ISL_855352_2020-08-03  301 TAGTAGTACTTTCTTTTGAACTTCTACATGCACCAGCAACTGTTTGTGGACCTAAAAAGTCTACTAATTTGGTTAAAAACAAATGTGTCAATTTCAACTTCAATGGTTTAACAGGCACAGGTGTTCTTACTGAGTCTAACAAAAAGTTTC  450
Hiro97618_03-08-2020                                    301 ......................................................................................................................................................  450
                                                            ******************************************************************************************************************************************************

hCoV-19_Japan_Hiro66017_2020_EPI_ISL_855345_2020-03-14  451 TGCCTTTCCAACAATTTGGCAGAGACATTGCTGACACTACTGATGCTGTCCGTGATCCACAGACACTTGAGATTCTTGACATTACACCATGTTCTTTTGGTGGTGTCAGTGTTATAACACCAGGAACAAATACTTCTAACCAGGTTGCTG  600
Hiro66017_14-03-2020                                    451 ......................................................................................................................................................  600

hCoV-19_Japan_Hiro99262_2020_EPI_ISL_855346_2020-04-11  451 TGCCTTTCCAACAATTTGGCAGAGACATTGCTGACACTACTGATGCTGTCCGTGATCCACAGACACTTGAGATTCTTGACATTACACCATGTTCTTTTGGTGGTGTCAGTGTTATAACACCAGGAACAAATACTTCTAACCAGGTTGCTG  600
Hiro99262_11-04-2020                                    451 ......................................................................................................................................................  600

hCoV-19_Japan_Hiro99268_2020_EPI_ISL_855347_2020-04-11  451 TGCCTTTCCAACAATTTGGCAGAGACATTGCTGACACTACTGATGCTGTCCGTGATCCACAGACACTTGAGATTCTTGACATTACACCATGTTCTTTTGGTGGTGTCAGTGTTATAACACCAGGAACAAATACTTCTAACCAGGTTGCTG  600
Hiro99268_11-04-2020                                    451 ......................................................................................................................................................  600

hCoV-19_Japan_Hiro94253_2020_EPI_ISL_855348_2020-04-20  451 TGCCTTTCCAACAATTTGGCAGAGACATTGCTGACACTACTGATGCTGTCCGTGATCCACAGACACTTGAGATTCTTGACATTACACCATGTTCTTTTGGTGGTGTCAGTGTTATAACACCAGGAACAAATACTTCTAACCAGGTTGCTG  600
Hiro94253_20-04-2020                                    451 ......................................................................................................................................................  600

hCoV-19_Japan_Hiro96977_2020_EPI_ISL_855349_2020-07-27  451 TGCCTTTCCAACAATTTGGCAGAGACATTGCTGACACTACTGATGCTGTCCGTGATCCACAGACACTTGAGATTCTTGACATTACACCATGTTCTTTTGGTGGTGTCAGTGTTATAACACCAGGAACAAATACTTCTAACCAGGTTGCTG  600
Hiro96977_27-07-2020                                    451 ......................................................................................................................................................  600

hCoV-19_Japan_Hiro97166_2020_EPI_ISL_855350_2020-07-29  451 TGCCTTTCCAACAATTTGGCAGAGACATTGCTGACACTACTGATGCTGTCCGTGATCCACAGACACTTGAGATTCTTGACATTACACCATGTTCTTTTGGTGGTGTCAGTGTTATAACACCAGGAACAAATACTTCTAACCAGGTTGCTG  600
Hiro97166_29-07-2020                                    451 ......................................................................................................................................................  600

hCoV-19_Japan_Hiro97237_2020_EPI_ISL_855351_2020-07-30  451 TGCCTTTCCAACAATTTGGCAGAGACATTGCTGACACTACTGATGCTGTCCGTGATCCACAGACACTTGAGATTCTTGACATTACACCATGTTCTTTTGGTGGTGTCAGTGTTATAACACCAGGAACAAATACTTCTAACCAGGTTGCTG  600
Hiro97237_30-07-2020                                    451 ......................................................................................................................................................  600

hCoV-19_Japan_Hiro97618_2020_EPI_ISL_855352_2020-08-03  451 TGCCTTTCCAACAATTTGGCAGAGACATTGCTGACACTACTGATGCTGTCCGTGATCCACAGACACTTGAGATTCTTGACATTACACCATGTTCTTTTGGTGGTGTCAGTGTTATAACACCAGGAACAAATACTTCTAACCAGGTTGCTG  600
Hiro97618_03-08-2020                                    451 ......................................................................................................................................................  600
                                                            ******************************************************************************************************************************************************

hCoV-19_Japan_Hiro66017_2020_EPI_ISL_855345_2020-03-14  601 TTCTTTATCAGGATGTTAACTGCACAGAAGTCCCT  635
Hiro66017_14-03-2020                                    601 ...................................  635

hCoV-19_Japan_Hiro99262_2020_EPI_ISL_855346_2020-04-11  601 TTCTTTATCAGGGTGTTAACTGCACAGAAGTCCCT  635
Hiro99262_11-04-2020                                    601 ...................................  635

hCoV-19_Japan_Hiro99268_2020_EPI_ISL_855347_2020-04-11  601 TTCTTTATCAGGGTGTTAACTGCACAGAAGTCCCT  635
Hiro99268_11-04-2020                                    601 ...................................  635

hCoV-19_Japan_Hiro94253_2020_EPI_ISL_855348_2020-04-20  601 TTCTTTATCAGGGTGTTAACTGCACAGAAGTCCCT  635
Hiro94253_20-04-2020                                    601 ...................................  635

hCoV-19_Japan_Hiro96977_2020_EPI_ISL_855349_2020-07-27  601 TTCTTTATCAGGGTGTTAACTGCACAGAAGTCCCT  635
Hiro96977_27-07-2020                                    601 ...................................  635

hCoV-19_Japan_Hiro97166_2020_EPI_ISL_855350_2020-07-29  601 TTCTTTATCAGGGTGTTAACTGCACAGAAGTCCCT  635
Hiro97166_29-07-2020                                    601 ...................................  635

hCoV-19_Japan_Hiro97237_2020_EPI_ISL_855351_2020-07-30  601 TTCTTTATCAGGGTGTTAACTGCACAGAAGTCCCT  635
Hiro97237_30-07-2020                                    601 ...................................  635

hCoV-19_Japan_Hiro97618_2020_EPI_ISL_855352_2020-08-03  601 TTCTTTATCAGGGTGTTAACTGCACAGAAGTCCCT  635
Hiro97618_03-08-2020                                    601 ...................................  635
                                                            ***********************************
```

Supplementary Figure S1: The validity of amplification and sequencing between Sanger Vs Next Generation Sequencing (NGS)
