# Supplementary material for: Exercising the Sanger Sequencing Strategy for Variants Screening and Full-Length Genome of SARS-CoV-2 Virus during Alpha, Delta, and Omicron Outbreaks in Hiroshima
Source: Viruses. 2022 Mar 30;14(4):720. doi: 10.3390/v14040720 (PMC9030034; doi:10.3390/v14040720)
Supplement: Supplementary file 1 [file viruses-14-00720-s001.zip › Supplementary Figure S2.pdf]

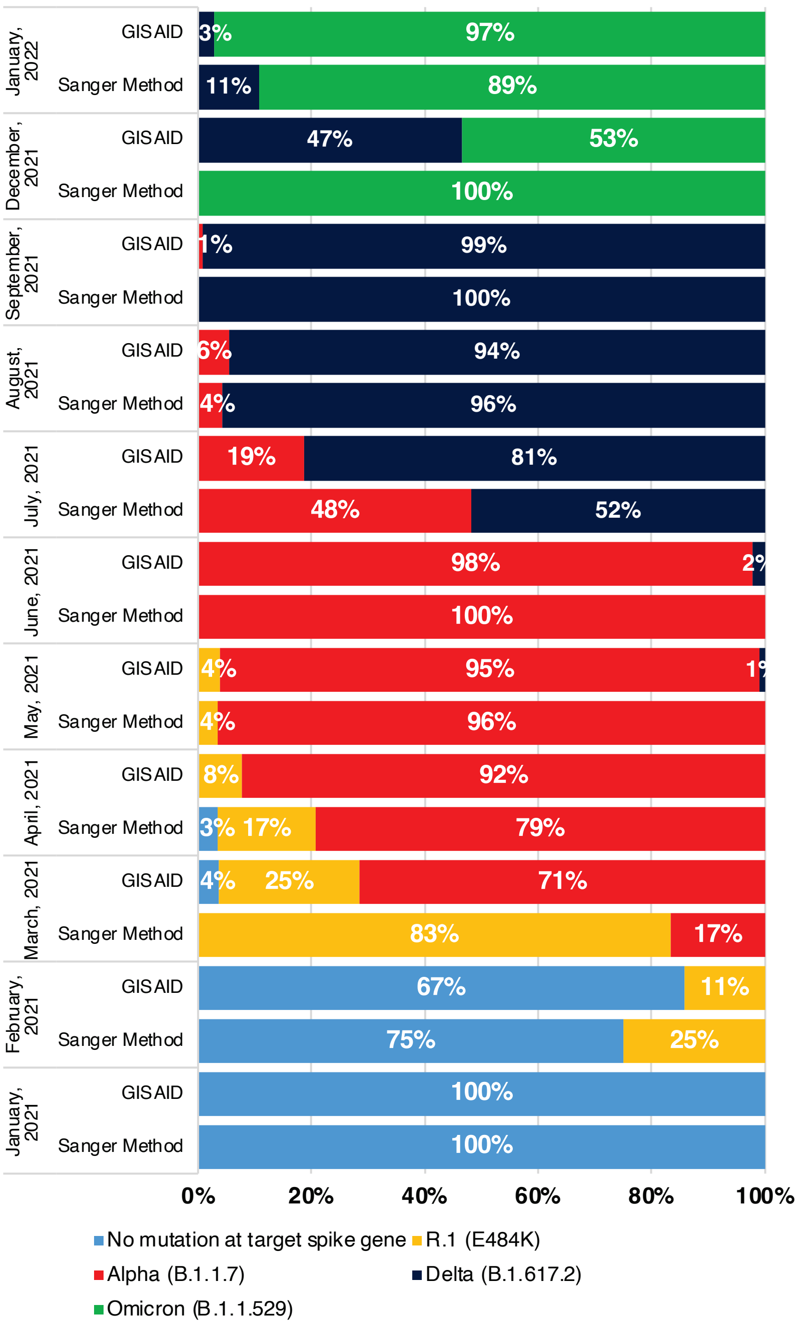

Supplementary Figure S2: The distribution of SARS-CoV-2 variants between Sanger and GISAID (January 2021-January 2022)
